# Supplementary material for: Intensive Behavioural and Pharmacological Treatment for Tobacco Dependence in Pregnant Women with Complex Psychosocial Challenges: A Case Report
Source: Int J Environ Res Public Health. 2020 Jul 2;17(13):4770. doi: 10.3390/ijerph17134770 (PMC7369832; doi:10.3390/ijerph17134770)
Supplement: Supplementary file 1 [file ijerph-17-04770-s001.pdf]

1. Patient Health Questionnaire (PHQ-9)

The Patient Health Questionnaire-9 [1] is a 9-item measure for assessing and monitoring depression severity against associated criteria from the DSM-V. Each item asks respondents to rate the severity of symptoms experienced over the previous two-week period on a 4-point Likert scale, scored as: not at all = 0, several days = 1, more than half the days = 2 and nearly every day = 3.Total scores range from 0 to 27 and can be interpreted as follows:

Table S1. Patient health questionnaire-9 scoring and interpretation.

SCORE RANGE	INTERPRETATION
0 - 4	minimal depression
5 - 9	mild depression
10 - 14	moderate depression
15 - 19	moderately severe depression
20 - 27	severe depression

2. Generalised Anxiety Disorder (GAD-7)

The Generalised Anxiety Disorder-7 [2] is a 7-item measure for assessing generalized anxiety disorder against associated criteria from the DSM-V. Each item asks respondents to rate the severity of symptoms experienced over the previous two-week period on a 4-point Likert scale, scored as: not at all = 0, several days = 1, more than half the days = 2 and nearly every day = 3. Total scores range from 0 to 21 and are interpreted as follows:

Table S2. Generalised Anxiety Disorder-7 scoring and interpretation.

SCORE RANGE	INTERPRETATION
0 - 4	minimal symptoms
5 - 9	mild symptoms
10 - 14	moderate symptoms
15 - 21	severe symptoms

3. Alcohol Use Disorders Identification Test for Consumption (AUDIT-C)

The Alcohol Use Disorders Identification Test for Consumption [3] is a 3-item alcohol screen that reliably identifies people who are hazardous drinkers or have active alcohol use disorders. Questions ask how often alcohol is consumed; how many standard drinks are consumed in a typical day; and how often more than 6 standard drinks are consumed on a single occasion. Each AUDIT-C question has 5 answer choices, with scores ranging from 0 to 4 and total scores ranging from 0 to 12. A score greater than or equal to 5 is considered adequate for identifying hazardous or high-risk drinkers.

4. Australian Treatment Outcomes Profile (ATOP)

The Australian Treatment Outcomes Profile [4] is a brief instrument that assesses a range of substance use, health and well-being measures for the previous 28-day period. Information gathered is used to monitor clinical outcomes in drug and alcohol treatment services across the domains of substance use, general health and wellbeing. Repeated administration of the ATOP at routine points over time provide an indication of client response to treatment interventions.

5. Childhood Trauma Questionnaire (CTQ)

The Childhood Trauma Questionnaire [5] is a brief self-report tool comprising of 28 items and five scales that assess the severity of different types of childhood trauma: Physical, Sexual, and

Emotional abuse, and Physical and Emotional neglect. The questionnaire also includes a Minimisation/Denial scale for individuals who may be under-reporting traumatic events.

A 5-point Likert scale is used for the responses which range from Never True to Very Often True, with numerical values from 1 to 5 (5 to 1 for reverse-scored items). Scale total scores range from 5 to 25, with higher scores indicating a greater severity of maltreatment. Scoring cutoff points for the five subscales are as follows:

Table S3. Childhood Trauma Questionnaire scoring and interpretation.

SCORE INTERPRETATION	EMOTIONAL ABUSE	PHYSICAL ABUSE	SEXUAL ABUSE	EMOTIONAL NEGLECT	PHYSICAL NEGLECT
**NONE OR MINIMAL**	5 - 8	5 - 7	5	5 - 9	5 - 7
**LOW TO MODERATE**	9 - 12	8 - 9	6 - 7	10 - 14	8 - 9
**MODERATE TO SEVERE**	13 - 15	10 - 12	8 - 12	15 - 17	10 - 12
**SEVERE TO EXTREME**	16 +	13 +	13 +	18 +	13 +

6. Fagerström Test for Nicotine Dependence

The Fagerström Test for Nicotine Dependence is a standard instrument for assessing the intensity of nicotine addiction. Six items evaluate the quantity of cigarette consumption, the compulsion to smoke, and nicotine dependence. The items are totalled to provide a score between 0 and 10, with higher scores indicating more intense physical dependence on nicotine. The scores can be interpreted according to the following cutoff points:

Table S4. Fagerström Test for Nicotine Dependence scoring and interpretation.

SCORE RANGE	INTERPRETATION
1 - 2	low dependence
5 - 7	moderate dependence
3 - 4	low to mod dependence
8 +	high dependence

References

1. Gilbody, S.; Richards, D.; Brealey, S.; Hewitt, C. Screening for depression in medical settings with the patient health questionnaire (PHQ): a diagnostic meta-analysis. *J. Gen. Intern. Med.* **2007,** *22* (11), 1596-1602; DOI:10.1007/s11606-007-0333-y.
2. Spitzer, R. L.; Kroenke, K.; Williams, J. B. W.; Löwe, B., A brief measure for assessing generalized anxiety disorder: the GAD-7. *JAMA Internal Medicine* **2006,** *166* (10), 1092-1097; DOI:10.1001/archinte.166.10.1092.
3. Bush, K.; Kivlahan, D. R.; McDonell, M. B.; Fihn, S. D.; Bradley, K. A., The AUDIT alcohol consumption questions (AUDIT-C): an effective brief screening test for problem drinking. *JAMA Internal Medicine* **1998,** *158* (16), 1789-1795; DOI:10.1001/archinte.158.16.1789.
4. Ryan, A.; Holmes, J.; Hunt, V.; Dunlop, A.; Mammen, K.; Holland, R.; Sutton, Y.; Sindhusake, D.; Rivas, G.; Lintzeris, N., Validation and implementation of the Australian Treatment Outcomes Profile in specialist drug and alcohol settings. *Drug Alcohol Rev.* **2014,** *33* (1), 33-42; DOI:10.1111/dar.12083.

5. Bernstein, D. P.; Ahluvalia, T.; Pogge, D.; Handelsman, L., Validity of the Childhood Trauma Questionnaire in an adolescent psychiatric population. *J. Am. Acad. Child Adolesc. Psychiatry* **1997,** *36* (3), 340-348; DOI:10.1097/00004583-199703000-00012.
